# Care trajectory management: A conceptual framework for formalizing emergent organisation in nursing practice

**DOI:** 10.1111/jonm.12645

**Published:** 2018-07-17

**Authors:** Davina Allen

**Affiliations:** ^1^ School of Healthcare Sciences Cardiff University Cardiff UK

**Keywords:** care trajectory management, emergent organisation, quality and safety

## Abstract

**Aim:**

To offer a new conceptual framework for formalizing nurses’ work in managing emergent organisation in health and social care.

**Background:**

Much health and social care requires continuous oversight and adjustments in response to contingencies. Nurses have an important role in managing these relationships.

**Evaluation:**

A longstanding programme of research on the social organisation of health and social care work provided the foundations for the article.

**Key issue:**

Nurses’ work in managing emergent organisation may be conceptualized as care trajectory management and factors contributing to trajectory complexity are explored.

**Conclusions:**

Care trajectory management is essential for the quality and safety of health and social care but poorly served by existing management frameworks.

**Implications for Nursing Management:**

Care trajectory management offers a conceptual framework for the development of new management structures to support an important but poorly supported element of nursing practice.

## 1 INTRODUCTION

This paper offers a new, evidence‐based conceptual framework for formalizing nurses’ work in managing “emergent organisation” in health and social care systems. While formal management approaches—pathways, standards and protocols—are effective mechanisms for coordinating care in many areas of service delivery, a significant proportion of health and social care work depends for its success on emergent organisation, that is, continuous oversight and on‐going negotiations in response to contingencies. Nurses have an important role in managing these arrangements, but while widely acknowledged anecdotally, this work lacks formal recognition and is poorly served by existing management systems. The quality and safety of health and social care depends on ensuring that all the necessary elements to meet patient needs are aligned in the right place and at the right time and as the complexity and intensity of health and social care continues to accelerate a failure to acknowledge the need to manage emergent organisation in achieving this aim is an important gap in existing organisational infrastructures.

This article draws together three components of a longstanding programme of research on the social organisation of health and social care: primary ethnographic research which examined in‐depth the organisational elements of the nursing role in a tertiary hospital in Wales (Allen, 2015); translational mobilization theory (TMT) (Allen, 2018; Allen & May, 2017), a generic sociological theory, arising from this empirical work and designed to describe and explain emergent projects of collective action in conditions of organisational complexity; and a body of “trajectory” studies examining the delivery and organisation of care (Allen, 2000, 2004; Allen, Griffiths, & Lyne, 2004a, 2004b). The manuscript proceeds as follows. First, it introduces the notion of emergent organisation in health and social care and describes the conditions that give rise to this. Second, it summarizes ethnographic research on the nursing contribution to emergent organisation (Allen, 2015) and makes the case for its formalization. Third, it presents a secondary analysis of the original study deploying TMT (Allen, 2018; Allen & May, 2017) to conceptualize the work of managing emergent organisation as care trajectory management. Fourth, it combines these insights with research on the organisation of health and social care work to explore some of the factors that contribute to trajectory complexity. Finally, it considers the implications and applications of formalizing emergent organisation and care trajectory management in health and social care.

## 2 EMERGENT ORGANISATION IN HEALTH AND SOCIAL CARE

Health and social care is arguably the most complex system of work in contemporary society. Patients receive input from different providers and these relationships are conditioned by differences in knowledge, occupational cultures, social worlds, power and prestige. Service delivery is characterized by action and knowledge that is distributed across time and space (Zerubavel, 1979); fragmented and multiple understandings of the patient (Mol, 2002); and staff that make largely independent contributions to care (Allen, 2015). Additionally, this complex system of work is embedded in a turbulent environment. Care organisations have less control over workflows than do other services and experience constant churn (Duffield et al., 2007) with the care of individuals having to be balanced with that of whole populations. Ineluctably “people work”, health and social care has a high degree of unpredictability—increasingly so in ageing populations with complex needs and comorbidities. Patients and their families interact with delivery processes: they are both producers and consumers of services.

Failures of coordination are well‐recognized threats to the quality and safety of care provision (Kobewka et al., 2016) and the proliferation of check lists, care pathways and protocols in recent years are an attempt to tame the complexity of delivery processes to mitigate these risks. These are not without value, but there will always be some elements of health care work that resist such attempts at rationalization and control and depend for their success on what I call “emergent organisation”, that is forms of organisation characterized by ongoing management and negotiation in response to exigencies. Although they do not use this term, Strauss et al. capture the phenomenon of emergent organisation powerfully in their classic study of the social organisation of medical work, where they compare the challenges of managing health care with the challenges that confront the river pilot in navigating the channels of the Mississippi:

[T]he river was tricky, changed its course slightly from day‐to‐day, so even an experienced, but inattentive pilot could run into grave difficulties; worse yet, sometimes the river drastically shifted in its bed for some miles into a new course. […] Some of the various contingencies may be anticipated, but only a portion of them may be relatively controllable, […] stemming as they do, not only from the illnesses themselves but from organizational sources. (Strauss, Fagerhaugh, & Suczet, 1985).

Nurses have a central, but relatively invisible, role in managing these relationships.

### 2.1 The nursing contribution to emergent organisation and the case for formalization

The work of 40 hospital‐based nurses in managing emergent organisation in health and social care was studied using ethnographic methods and conceptualized as “organising work” (Allen, 2015). Organising work refers to those everyday elements of nursing practice concerned with the coordination and organisation of patient care. It is related to but distinct from direct patient care and nursing management. Whereas the former is patient‐focused and the latter is primarily unit‐focused, organising work is “care trajectory”‐focused. Derived from Strauss, Fagerhaugh, and Suczet (1985) classic “illness trajectory” concept, “care trajectory” refers to “the unfolding of patients” health and social care needs, the total organisation of work associated with meeting those needs, plus the impact on those involved with that work and its organisation’ (Allen et al., 2004a). The study highlighted the ubiquity of emergent organisation in contemporary health care systems and the central role of nurses’ in trajectory management:

“Their location in the sites of care and at critical departmental and organisational interfaces casts nurses in a pivotal role in mediating the relationships between the heterogeneous actors through which patient and population needs are addressed. Through four inter‐related domains of practice nurses function as obligatory passage points in hospital orders: creating the working knowledge that supports care delivery; articulating the configurations of socio‐material actors required to meet individual needs; matching people with beds and supporting patient flows; and parsing patient identities to secure transfers of care. Not only is this work an essential driver of action, it also operates as a powerful countervailing force to the centrifugal tendencies inherent in healthcare organisations which, for all their gloss of order and rationality, are actually very loose arrangements”. (Allen, 2015, p. 132).

Since its emergence as a formally recognized occupation in the mid nineteenth century, nursing has always entailed an organisational component. Nightingale considered the “art of nursing” to include responsibility for creating the conditions to promote healing and health and many of her interventions were designed to improve the organisation of care and the material environment. In recent history, however, the profession's self‐understanding has neglected this facet of the nursing role, foregrounding the direct rather than indirect dimensions of patient care. The emergence of new public management in the 1980 s (Hood, 1995) saw nursing models of organisation increasingly replaced by those of general management (Strong & Robinson, 1990) which emphasize audit, rational planning and standardization as the desired means for achieving organisational objectives (Power, 1997). Nurses have led the implementation of these new technologies and have achieved some notable improvements in health care quality and safety (Allen, 2010a, 2010b; Morrow, Robert, Maben, & Griffiths, 2012). However, the attendant preoccupation with “measure and manage” (Waring, 2007) heralded by such approaches has rendered invisible emergent organisation and the work of nurses in managing trajectories of care.

Strauss et al. (1985) deployed the Mississippi River Pilot metaphor to capture how health care work had been “radically and irrevocably” altered by the prevalence of chronic diseases and the specialization of technologies developed to manage them. In the intervening 30 years these impulses have continued unabated and have been overlaid with resource pressures, coexistent morbidities, and accumulative complexity (May et al., 2016), producing increased acuity and accelerated throughput in the acute sector (Duffield et al., 2007) and a redistribution of care (Exley & Allen, 2007) and treatment (May, 2013) in the community. Thus, while some areas of health and social care are increasingly routinized, there remain great swathes of activity where service delivery depends on flexible responses to unfolding needs and contingencies. Given that breakdowns of coordination are a major contributor to failures in quality and safety (Kobewka et al., 2016; Waring, Bishop, & Marshall, 2016; Waring, McDonald, & Harrison, 2006), there is a compelling case for the formalization of emergent organisation.

## 3 CARE TRAJECTORY MANAGEMENT: A FRAMEWORK FOR FORMALIZING THE WORK OF NURSES IN MANAGING EMERGENT ORGANISATION IN HEALTH CARE SYSTEMS

This section introduces care trajectory management as a conceptual framework for formalizing nurses’ work in supporting emergent organisation in health and social care systems. The framework has been developed from a secondary analysis of the original ethnographic research (Allen, 2015) drawing on translational mobilization theory (TMT) (Allen, 2004, 2018; Allen & May, 2017). TMT is a generic sociological theory of emergent organisation and has three components. The “project” is the primary unit of analysis; it provides a frame for understanding the relationships in a trajectory of care. The “strategic action field” defines the contexts in which projects (trajectories) are mobilized and which furnish the resources (structures, organising logics, interpretative repertoires, materials and technologies) through which action is organised and managed. “Mechanisms” direct attention to *how* projects of collective action are mobilized. These include: object formation (how actors construct the focus of their activity); translation (how these different understandings are shared and differing viewpoints accommodated); reflexive monitoring (how actors maintain project awareness); articulation work (how the different elements in a project are aligned to support action and decision making); and sensemaking (how actors comprehend and create order in work).

### 3.1 Care trajectory management

Care trajectory management can be conceptualized as comprising three components: trajectory awareness (practices that maintain awareness of trajectories of care); trajectory working knowledge (practices that support information sharing to allow care to progress); and trajectory articulation (practices that ensure all the elements necessary to meet patient needs—expertise, materials, information—are aligned in the right place and at the right time). This work is illustrated below by reference to the work of hospital nurses.

### 3.2 Trajectory awareness

“Knowing exactly what's going on everywhere”. [Senior Nurse].

Trajectory awareness refers to the work of maintaining oversight of trajectories of care as they evolve in time and space. For much of the time, facts and understanding pertinent to an individual's care are dispersed throughout a diverse network of professionals, communities, artefacts and information systems (Ellingsen & Monteiro, 2003) and so arrangements must be in place to enable participants to pool resources and negotiate to accomplish their tasks. In the hospital context, nurses fulfil this function through the generation and maintenance of “trajectory summaries”. These are narratives that encapsulate the overall status of patient trajectories and are typically initiated when patients are admitted to a service, circulated through the nursing handover and then regularly updated as trajectories evolve. Maintaining trajectory narratives involves work. Reflexive monitoring (May & Finch, 2009) refers to the processes through which nurses review an individual's care and treatment, the status of the clinical environment and the organisation, and assess the implications of this relationship for trajectory management. Sensemaking (Weick, 1995) refers to the work that nurses undertake to create order from the different information sources related to patient care. They make decisions about what information to take note of and what to ignore, reach judgements about the accuracy of different knowledge sources, and resolve inconsistencies. Through their reflexive monitoring and sense‐making work nurses create the awareness that supports care trajectory management.

### 3.3 Trajectory working knowledge

“We're the link; they tell us and then we tell everyone else!” [Senior Nurse].

“Working knowledge” refers to the translational work that creates the information flows necessary for the on‐going organisation of trajectories. Derived from actor network theory (Latour, 2005), in TMT “translation” refers to the practices that enable differing viewpoints and multiple interests within a care trajectory to be accommodated in order to enable concerted action. Good communication in health and social care is typically understood as a case of ensuring the comprehensiveness of information. The work of hospital nurses reveals that in practice, successful trajectory management depends less on the comprehensiveness of information and more on ensuring that the right information is shared for the purposes at hand (Allen, 2015). Nurses draw on their relational knowledge of trajectory actors and select out the relevant elements of the story. The centrality of translational processes to creating working knowledge is brought into sharp relief in the work nurses do managing transfers of care across departmental and/or organisational boundaries. This involves complex translational processes in order for a patient to be safely transferred from one context to another and which is too easy to trivialize as paperwork (Allen, 2015).

### 3.4 Trajectory articulation

“Nurses run the place. […] That requires anticipating people's needs and constantly being two steps ahead” [Senior Nurse].

Trajectory articulation refers to the practices through which trajectory elements are aligned in time and space. The original concept is derived from Strauss et al. (1985) who deployed the term to refer to the secondary work processes necessary to align trajectory activity and to ensure “that the staff”s collective efforts add up to more than discrete and conflicting bits of accomplished work’ (p. 151). Health and social care is complex and decisions must be taken about what should be done, by whom, when, where and with what materials. The more elements involved, the more challenging this becomes. Moreover, because health and social care is distributed work, it is rare that all providers come together to coordinate their activity. Nurses undertake three different kinds of articulation work. “Temporal articulation” is undertaken to ensure things take place at the right time and in the right order. Here nurses draw on their organisational knowledge and understanding of processes and procedures in order to anticipate need and plan. “Integrative articulation” is designed to ensure decision‐making is joined up. When largely independent actors interact around the patient, decisions that seem reasonable in isolation can be problematic in the context of a wider trajectory of care—so nurses have an important role in identifying and addressing these potential dangers. “Material articulation” aims to ensure the availability of materials and resources to support care. This is not a mundane consideration; lack of equipment is an important cause of safety incidents. In the acute sector, “bed management” is an important form of material articulation. The “bed” is associated with a whole host of resources: people, knowledge, space and technology. Placing someone in the most appropriate bed helps ensure the resources needed to meet their needs are available (Allen, 2014).

### 3.5 Care trajectory complexity

Consistent with the concept of a care trajectory, sources of trajectory complexity arise not only from the uncertainty of attending to injury and disease, but also from the division of labour, the turbulence of the work environment, and biographic and psycho‐social considerations relating to patients, kin and staff. This section draws together findings from the study of nursing (Allen, 2015) with a body of work on the management of patient trajectories (Allen, 2000, 2004; Allen et al., 2004a, 2004b) to begin to explore some of the factors that impact on trajectory complexity.

At a fundamental level, diagnostic ambiguity makes trajectory management more complex as does the existence of co‐morbidities as this delimits the applicability of standardized care pathways and increases the uncertainty of care and treatment. Indeed, a recent Canadian hospital's institution‐wide mortality review, reports that the most important quality gaps in this organisation arose from the failure of health care workers to coordinate their efforts around two key goals: treatment plans and diagnosis (Kobewka et al., 2016). Disagreements between the health care team and family carers may also compound trajectory management (Allen, 2000) but while a divergence of views can make trajectory management more challenging, this does not necessarily lead to poorer outcomes for patients. Our study of stroke rehabilitation showed how conflicts between members of the health and social care team increased trajectory complexity and prolonged the hospital admission of Edward (Allen, Griffiths, & Lyne, 2004b) but in the longer term ensured a better outcome for the family as it allowed the exploration of a plurality of perspectives. In this study we highlighted how pressures on health and social care staff acted as a strong incentive to manage out complexity rather than working through it in the interests of patients.

Trajectory complexity is also influenced by the number of actors involved. Each additional actor adds a different perspective that must be aligned and extends the distribution of trajectory work in social time and space. Relationships between trajectory actors are critical. The study of nurses’ organising work highlighted that nurses’ familiarity with providers impacted on the ease with which trajectory management work could be accomplished, as did the inclusion of transient staff who lacked familiarity with organisational processes. Relatedly, the growing number of “outliers”, that is patients placed in beds outside of the service responsible for their care, made trajectory management more challenging. An anticipatory plan was problematic as nurses did not have access to the relevant organisation routines and standards and it was more demanding to progress care when interacting with unfamiliar clinical teams. Evidence suggests “outlying” patients impacts negatively on patient outcomes (Bai et al., 2018).

Psycho‐social factors also have implications for trajectory management. For example, research highlighted how the biographical disruption (Bury, 1982) experienced by patients who had suffered a first acute stroke and their families had implications for the ease with which on‐going care arrangements could be negotiated (Allen et al., 2004b). This study also showed how socio‐economic factors impacted significantly on managing the trajectory from hospital to home: families who had access to private finance were able to progress their on‐going care arrangements; whereas families that were dependent on publicly funded services could not and became designated as “bed blockers” (Allen et al., 2004a). The study of nursing also revealed how disputes between agencies over funding arrangements can complicate trajectory management.

There are undoubtedly other sources of complexity that might be listed here and the specific factors that impact on trajectory complexity in a given context—acute, community, primary care—and for different populations—adult, children, mental health, older people—will vary. My aim here is to draw on the research with which I am familiar to explore some of the possible sources of trajectory complexity to draw out the logic of formalizing emergent organisation for health and social care.

## 4 IMPLICATIONS AND APPLICATIONS

The formalization of emergent organisation has a number of implications for health and social care policy and practice.

First, emergent organisation is not well served by existing management technologies. Many of the nurses in the original study had developed their own methods and tools to support their practices. While reflecting the logic of their organising work, these operated under the radar of formal organisational processes and, lacking legitimacy, were not integrated into management and information systems. Formalizing care trajectory management opens up the possibility of developing a systematic approach to assessing care trajectory complexity in order to build this awareness into workforce planning and service delivery and organisation. This should not be taken to imply the possibility of rationalizing emergent organisation, but rather the aspiration to facilitate more proactive approaches to anticipating and managing emergence and complexity which may reduce the likelihood of what Strauss et al. call “cumulative mess trajectories” with all the attendant risks to quality and safety.

“[E]fforts to keep the trajectory on a more or less controllable course look somewhat gyroscopic. Like the instrument, they do not necessarily spin upright but, meeting contingencies, they may swing off dead centre—off course—for a while before getting righted again, but only perhaps to repeat going awry one or more times before the game is over. Sometimes, though, the trajectory game finishes with a total collapse of control, quite like the gyroscope falling to the ground”. (Strauss et al., 1985, p. 20).

Formalizing emergent organisation also has implications for the development of information systems. The original study revealed that assembling the knowledge to inform trajectory narratives was onerous; the contemporary medical record is increasingly fragmented with patient information distributed across different artefacts and technologies, both paper‐based and electronic. As health care systems increasingly embrace digital technologies, this opens up the possibility of automated generation of trajectory summaries available to nurses in handheld devices and which can be readily updated. Furthermore, many of the nurses in my original study (Allen, 2015) kept personal notebooks including local information and knowledge to support organising work. They could easily become deskilled, however, if they moved outside of the environments with which they were familiar or were required to care for patients whose care brought them into contact with new structures and actors with unknown work purposes and organising logics. This raises the question about how this local knowledge might be shared.

Second, although a relatively invisible but highly skilled element of the nursing role, care trajectory management does not feature in assessments of safe staffing. Formalizing care trajectory management has the potential to inform the development of workforce planning tools that could be used to systematically assess the volume and complexity of care trajectory management work in clinical areas.

Third, while nurses’ care trajectory management makes an important contribution to the quality and safety of patient care, nurses have uncertain authority in performing this aspect of their role. Formalization would go some way to overcoming the organisational hierarchies and power imbalances that make this work more challenging and would confer upon others, the obligation to orient their own practices to such arrangements.

Fourth, nurses have a central role in care trajectory management, but it is not an exclusively nursing activity. In different contexts it may be more evenly distributed between actors, in others, it might fall disproportionately to particular occupational groups or technologies, and increasingly in the community this responsibility falls on family carers. Formalization is the first step towards developing a more sophisticated understanding of how emergent organisation is achieved in different contexts and the technologies and resources that might facilitate this work irrespective of who is assigned this responsibility.

## 5 CONCLUSION

This paper has made the case for formalizing emergent organisation and the work of care trajectory management in health and social care and has reflected on some of the implications of such a strategy. Creating an organisational space and infrastructure for models of organisation and management founded on flexibility, negotiations and contingency in the face of dominant neoliberal management logics in health and social care will not be easy, but it is essential if the quality and safety of health and social care is to be assured. This is an urgent and essential leadership role for nurse managers. Paradoxically as health and social care is increasingly organised through management models that emphasize standardization and rationalization, a growing number of service users present with non‐standard and uncertain needs.

## ETHICAL APPROVAL

The paper is a secondary analysis of previously published work and does not require ethics approval.
